# Nutrition and Environmental Pollution Extension Curriculum Improved Diet-Related Behaviors and Environmental Health Literacy

**DOI:** 10.1177/1178630219836992

**Published:** 2019-03-29

**Authors:** Dawn Brewer, Hannah Bellamy, Anna Hoover, Annie Koempel, Lisa Gaetke

**Affiliations:** 1 Department of Dietetics and Human Nutrition, University of Kentucky, Lexington, KY, USA; 2 Department of Epidemiology, University of Kentucky, Lexington, KY, USA; 3 College of Public Health, University of Kentucky Lexington, KY, USA

**Keywords:** Nutrition, environmental health, health education

## Abstract

Kentucky experiences some of the nation’s worst health outcomes related to obesity, diabetes, high blood pressure, and other age-related chronic diseases linked with oxidative stress and inflammation, which in turn are associated with poor diet, lack of physical activity, and exposure to certain environmental pollutants. In the Commonwealth, deteriorating infrastructure, inappropriate waste disposal, and potential occupational injury related to mining, agriculture, and other regionally important industries exacerbate the need for residents to have basic knowledge of potential environmental health threats. Unfortunately, community-level understanding of the complex connections between environmental exposures and health is limited, with many Kentuckians unaware that the Commonwealth is home to 13 hazardous waste sites included in the United States Environmental Protection Agency Superfund National Priorities List (NPL). The NPL highlights priority sites for long-term remedial action to reduce environmental contaminants. To enhance the understanding of environmental health and protective actions, the University of Kentucky Superfund Research Center Community Engagement Core developed a 9-lesson extension curriculum “Body Balance: Protect Your Body from Pollution with a Healthy Lifestyle” (Body Balance) and partnered with Kentucky’s Family and Consumer Sciences (FCS) Cooperative Extension Service to pilot the curriculum in Kentucky communities. FCS agents in 4 Kentucky counties delivered the *Body Balance* pilot study (18-31 participants per lesson). Pre- and post-lesson questionnaires revealed increased knowledge and awareness of the effects of environmental pollution on health and the protective role of dietary strategies. Focus group participants (n = 18) self-reported positive behavior changes because of increases in knowledge and leadership from their FCS agent. The *Body Balance* curriculum appeared to be a promising mechanism for raising environmental health and diet knowledge, as well as for promoting positive behavior changes among white, middle/older-aged women in rural Kentucky communities.

## Introduction

Many age-related chronic diseases are associated with an underlying presence of oxidative stress and inflammation.^1^ Healthy dietary practices and increased physical activity have long been recognized to prevent or reduce the progression of chronic diseases.^2^ With the risk of developing chronic diseases closely linked to certain environment factors,^3^ the University of Kentucky Superfund Research Center (UK-SRC) explores the complex relationships among chronic disease, inflammation, and the environment. Specifically, the UK-SRC studies the hypothesis that unhealthy dietary practices exacerbate a person’s vulnerability to the negative health effects of environmental pollution. UK-SRC research findings to date have shown that nutrition differentially affects environmental pollution-driven oxidative stress and inflammation.^4,5^

Although many traditional studies of this relationship focus on food ingestion as a potential route of exposure to contaminants that contribute to chronic disease and acute illnesses,^6-8^ UK-SRC also recognizes foodstuffs as providers of key nutrients that can modulate environmental insults in a positive or negative manner.^9^ For example, phytonutrients found in plant matter are believed to protect against chronic diseases through their anti-inflammatory and antioxidant properties.^10^ Phytonutrients have been found to reduce toxicological insults associated with environmental pollutants.^4^ In contrast, certain nutrients can hasten the development of chronic diseases.^11-13^ Furthermore, food itself potentially serves as a point of chemical exposures because contamination can occur at several points during processing, resulting in the presence of potentially toxic compounds in foods.^14^ These contaminants can then be passed to humans via the food chain, either directly through human consumption, residue of contaminated fruits or vegetables, or consumption of meat and dairy foods from animals with contaminants stored in their fat tissues.^14^ The UK-SRC Community Engagement Core (CEC) supports the Center’s work by disseminating research findings, engaging in bi-directional communication with affected or concerned communities, and implementing appropriate nutrition-related activities to increase awareness and knowledge. In doing so, the CEC promotes behavior changes that can help modulate the poor health outcomes linked to environmental pollution.

Exposure to environmental pollution is a concern in Kentucky, which has recognized approximately 1000 contaminated sites^15^ in addition to 13 Superfund sites that are listed on the United States Environmental Protection Agency (US EPA) National Priorities List (NPL). Kentucky ranks 11th among US states for most total chemical releases per square mile.^16^ Moreover, 422 Kentucky facilities listed in the US EPA Toxic Release Inventory (TRI) reported a release of 53.39 million pounds of chemicals into the environment, including releases into air (22.1 million pounds), water (7.3 million pounds), and placement in on-site or off-site managed land disposal units.^17,18^ The top 5 chemicals released into the air include sulfuric acid aerosols, methanol, hydrochloric acid aerosols, toluene, and ammonia. Of these chemicals, methanol, hydrochloric acid, and toluene have been deemed hazardous for health because they cause or are suspected of causing cancer, birth defects, or other serious harms.^19^ The top 5 chemicals released into water included nitrate compounds, manganese compounds, ammonia, methanol, and barium compounds. Nitrate and barium fall under the US EPA-regulated National Primary Drinking Water Regulations designed to protect public health by enforcing maximum concentration level standards limiting the presence of certain compounds in public water systems.^20^ Kentucky, however, is estimated to have more than 200 000 water wells that are not monitored for contaminants^21^ or regulated to limit the presence of contaminants.^22^ To compound the issue, Kentucky surface and groundwater supplies are susceptible to undesirable levels of both natural and anthropogenic pollution. Pollutants include iron, manganese, barium, selenium, hydrogen sulfide, and salt; bacteria and nitrate/nitrogen from various sources including sewage; organic chemicals that are by-products of water disinfection (trihalomethanes); and such industrial solvents as trichloroethylene,^23^ which EPA recognizes as a known carcinogen. In Kentucky, non-point pollution sources pollute 3.5 times as many miles of streams as point sources. The top non-point sources of pollution in Kentucky include mining (31%), agriculture (29%), land disposal/septic systems (20%), and urban runoff (10%).^21^

Recently, the field of Environmental Health Literacy (EHL) has emerged to promote a better understanding of the links between environmental exposures and human health.^24^ Social scientists working in EHL assess individual and community knowledge of complex connections between specific environmental contaminants, illness, and health-protective actions.^24^ After identifying context-specific knowledge gaps, EHL researchers and practitioners strive to increase understanding of environmental health issues among at-risk individuals through a variety of strategies, including enhanced report-back of environmental exposure results^25,26^ and even arts-informed strategies for fostering knowledge-sharing.^27^ Many of these approaches are situated within theoretical frameworks derived from the education field. For example, Bloom’s taxonomy has been adapted for EHL to indicate that the skills and knowledge needed to be environmental health literate are context-specific. Although some individuals and communities might simply need to recognize that a substance is a potential threat to avoid exposure, others might need to be able to create action plans that reduce the community-wide likelihood of exposures and/or to improve individual health outcomes after an exposure has happened.^28^ Regardless of the approach or theoretical framework underlying EHL activities, researchers and practitioners working in the field share an understanding that enhancing EHL can help move individuals and communities to take health-protective actions.^24^

Regarding health, Kentucky ranks near the bottom of all US states in many key health indicators, including obesity, chronic diseases, and poor diet.^29^ Although high rates of physical inactivity and smoking increase risk of such illnesses, so do such social determinants of health (SDOH) as lack of education and poverty. Lower levels of baseline health may increase susceptibility to the detrimental health effects of environmental pollution^9,30^ (Table 1) while SDOH can widen the knowledge gaps that impede health-protective actions.

**Table 1.** Health outcomes and socioeconomic factors of counties participating in Body Balance pilot study.

	Knox County 1	Pike County 2	Todd County 3	Washington	Kentucky County 4	United States
Geographic location in Kentucky	Southeast	East	Southwest	Central		
Rural-urban continuum category (Code)^a^	Non-metro, urban population of 2500 to 19 000, not adjacent to a metro area (7)	Non-metro, urban population of 2500 to 19 000, not adjacent to a metro area (7)	Non-metro, completely rural or less than 2500 urban population, adjacent to a metro area (8)	Non-metro, completely rural or less than 2500 urban population, not adjacent to a metro area (9)		
2017 population estimates^31^	31 227	58 883	12 243	12 126	4 454 189	
Health outcomes (rank among 120 KY counties)^b^	107	106	44	14		
Cancer deaths (all cancers, age-adjusted rate per 100 000 population)^32,33^	212	233	168	174	198	156
Prevalence of diabetes (adults)^32,34^	17%	21%	12%	16%	13%	11%
Heart disease deaths (per 100 000 population)^32,35^	217	245	211	182	200	166
Length of life (rank)^b^	105	107	40	5		
Health behaviors (rank)^b^	120	83	46	27		
Adult smoking^b^	29%	22%	21%	20%	24%	14%
Adult obesity^b^	43%	40%	35%	33%	34%	26%
Food environment index^b^	6.6	7.5	8.2	7.7	7.0	8.6
Physical inactivity^b^	33%	35%	39%	30%	28%	20%
Clinical care (rank)^b^	102	73	104	48		
Social and economic factors (rank)^b^	106	108	20	14		
Some college^b^	40%	47%	48%	48%	60%	72%
Unemployment^b^	7.9%	10.8%	4.2%	4.1%	5.0%	3.2%
Children in poverty^b^	47%	42%	28%	23%	24%	12%
Air pollution (particulate matter µg/m^3^)^b^	9.7	9.7	10.2	9.8	10.0	6.7
Drinking water violations^b^	No	Yes	No	No		

a USDA Economic Research Service (ERS) Rural-Urban Continuum Codes form a classification scheme that distinguishes metropolitan (metro) counties by the population size of their metro area, and non-metropolitan (non-metro) counties by degree of urbanization and adjacency to a metro area or areas.^36^

b Rank of a particular county of Kentucky’s 120 counties. The lowest score is associated with best health and the highest score with worst health.^37^

Prior research showing that EHL among Kentuckians is low,^38^ along with the prevalence of poor health outcomes and heightened exposure risks, pointed to a need for curricula designed to increase knowledge and awareness of protective actions that may mitigate exposure-linked negative health outcomes. In response, the CEC developed a 9-lesson extension curriculum titled “*Body Balance: Protect Your Body from Pollution with a Healthy Lifestyle*” (*Body Balance*). The *Body Balance* curriculum highlights dietary and other lifestyle strategies to reduce exposures and/or protect against environmental pollution, including risks related to food contamination.

The research team engaged Kentucky’s well-established Family and Consumer Sciences (FCS) Cooperative Extension System as a key *Body Balance* implementation partner. FCS Extension helps people make informed decisions about their well-being, relationships, and resources to achieve optimal quality of life.^39^ The CEC regularly partners with FCS to disseminate healthy lifestyle and environmental pollution messages to Kentucky residents. Extension is strategically positioned to influence all 5 spheres of the Social-Ecological Model (a systems approach to health promotion) for behavior change—individual, interpersonal, organizational, community, and systems or policy.^40,41^ Implementing multiple changes at various levels of the Social-Ecological Model for behavior change has been shown to be effective in improving eating and physical activity behaviors.^42^ Therefore, the CEC leveraged the educational activities of FCS Extension to directly address individual, organizational, community, and system factors by having agents incorporate a nutrition and environmental pollution–focused education series into their programming. The *Body Balance* curriculum itself directly addresses individual and interpersonal factors of the Social-Ecological model by offering easy nutrition-related behavior choices that have the potential to affect participants as well as their families and friends. By incorporating an FCS Extension agent to deliver the curriculum, program implementation further addresses organizational, community, and system factors by ensuring delivery from an integral, trusted member of the community who has the potential to influence collective decisions and norms related to nutrition behavior.

For this pilot study, the CEC and FCS Extension partnered to assess whether curriculum delivery improved EHL levels and self-reported protective food-related behaviors among community members who participated in the *Body Balance* curriculum.

## Methods

### Development of lesson series

The *Body Balance* lesson series consisted of 9 nutrition-based lessons. A needs assessment conducted in a previous study,^38^ as well as discussions with UK-FCS agents and members of the UK-SRC Research and Translation Core (RTC), identified potential lesson topics. The series was developed for Kentuckians with a focus on community participants in FCS programs, which primarily comprise middle-aged to older adult white women.

### Selection of participating counties

The Assistant Director for UK’s FCS Field Programs assisted the research team in identifying 5 FCS agents as potential partners to pilot the *Body Balance* lesson series in their respective counties. In March, investigators described the project via email to 5 agents; ultimately 4 agents representing 4 counties agreed to deliver the *Body Balance* curriculum to the target audience of their community members. The FCS Extension program offered *Body Balance* in a manner consistent with other program offerings in their respective counties. The 4 counties included in this study were located in east (County 1), southeast (County 2), southwest (County 3), and central (County 4) Kentucky, representing 4 of the Commonwealth’s 7 county Extension districts (Table 1). The economies of the 4 participating counties are supported by industries associated with the release of pollutants into the environment.^43^ The two Eastern Kentucky counties are located in the coalfields of a region with a well-documented history of fossil fuel extraction; County 3 predominantly supports tobacco farming and other agricultural endeavors, as well as manufacturing industries;^44^ and County 4 also supports agriculture and manufacturing.^45^

Studies in Appalachian Counties of Eastern Kentucky have found increased levels of sulfur dioxide and other acidic particles in air samples,^46,47^ while community members have voiced water quality concerns.^48^ County 3 features both karst terrain and agricultural land-use, with the Groundwater Branch of the Kentucky Division of Water (KDOW) identifying areas of moderate to high sensitivity to groundwater pollution,^49^ consistent with the expectation that karst drainage is especially sensitive to agricultural non-point-source pollution from fertilizers, pesticides, and herbicides.^49^ The Groundwater Branch of KDOW similarly reported that County 4 has areas of moderate to high sensitivity to groundwater pollution.^49^

### Recruitment of community participants

This pilot study deployed convenience sampling, partnering with FCS agents to recruit community members via standard advertising strategies used for extension lesson series. Although the process varied across counties, recruitment channels included radio and newspaper advertisements, FCS Extension newsletters, flyers, Facebook posts, and word-of-mouth. Following recruitment, agents delivered *Body Balance* lessons to interested community members over approximately 3 months during the summer. County Extension offices hosted all lessons and focus groups for that county. The University of Kentucky Institutional Review Board approved all study activities.

### Lesson series evaluation

#### Questionnaires

The community members who participated in *Body Balance* lessons completed pre- and post-questionnaires for each individual lesson to assess changes in awareness and knowledge. A range of 18 to 31 total participants attended a particular lesson as they were presented in each county. Each questionnaire included 3 questions aligned with lesson content (Table 2). Demographic information collected included self-reported age, weight, height, sex, race, marital status, and highest level of education.

**Table 2.** Open-ended focus group questions.

I. To get our conversation started, we’re going to do an activity. I have 2 posters, with one phrase for each. Let me know what this phrase means to you. 1. What comes to your mind when someone mentions a “healthy lifestyle”? 2. What is “environmental pollution” to you?
II. Do you feel you are exposed to pollution? Think about what kind and how often. 1. What was your level of concern about pollution before and after the lessons? On a scale of 1-5, 1 being not concerned at all and 5 being very concerned, think back to what you would rate your level of concern before Body Balance? (wait a minute). Today, what would you rate your level of concern? 2. By a show of hands, who’s level of concern stayed the same? Increased? Decreased? 3. Do you have any thoughts on why your level of concern changed or didn’t change?
III. Has it ever crossed your mind that lifestyle choices can change how environmental pollution affects the body? If so, when did you start thinking about this? 1. Did you make any lifestyle changes as a result of the Body Balance lessons? If so, what changes? 2. How long did you continue the change? 3. Thinking back, why did you decide to make that specific change? 4. Are there any lifestyle changes you wanted to make but felt like you couldn’t? What kept you from making the change?
IV. What did you like about the program? 1. What was your favorite lesson? 2. Did you share any of the information you learned with your friends or family? What did you share?

#### Focus groups

Participants from 3 of the 4 participating counties agreed to participate in focus group discussions of the *Body Balance* curriculum, with participants in the fourth county opting out in favor of more informal, social extension activities. A total of 18 participants (range of 4-8 participants per group) took part in one of 3 focus groups, with their respective agent present, that was held in November. Each focus group lasted approximately 1 hour. Sessions were audio recorded, and field notes were taken. A graduate student moderated focus groups with support from a research assistant and 2 student observers. The moderator and research assistant were both Registered Dietitians (RD) who had prior training in focus group research.

The research team developed the focus group interview protocol to examine their hypothesis that *Body Balance* participants would increase their knowledge (EHL) of the protective impact of healthy lifestyle behaviors on health outcomes related to environmental exposures. The team further hypothesized that participants would self-report positive behavior changes based on improved knowledge of nutrition and environmental pollution. The interview guide was reviewed by 2 other RDs within the CEC and members of the RTC, including an expert in environmental health risk communication and 2 experts in assessing environmental pollution. Researchers pilot-tested the draft interview guide with 2 women of similar demographic characteristics as community members in our study who participated in the *Body Balance* lessons. Adjustments were made based on feedback (Table 2).

#### Data analysis

Questionnaire data were analyzed using SAS (v.9.4). Researchers calculated descriptive statistics for demographics, including frequencies, means, and standard deviations. Pre- and post-questionnaire categorical variables were compared within and between groups using McNemar’s test. Differences were considered statistically significant at *P* ⩽ 0.05.

The graduate student researcher and 2 undergraduate students transcribed verbatim the audio recorded focus group discussions. The graduate student moderator reviewed transcripts to ensure accuracy. Two independent researchers read and coded each transcript using axial coding, subsequently comparing findings and resolving discrepancies.^50^ Researchers developed themes and concepts using a deductive approach. Codes represented concepts specifically addressed in interview questions, as well as concepts that emerged during the focus group.

## Results

Total lesson attendance across the 4 counties ranged from 18 to 31 people attending a particular lesson with participants attending an average of 4.2 ± 3.0 lessons. The average age of lesson series participants was 62.2 ± 17.9 years: 92% women; 86% white; 83.8% reporting being single, divorced, or widowed; and 20% having less than a high school diploma, 45.5% with a high school diploma, and 34.5% with some college or a college degree (data not shown). There were no differences in baseline knowledge or change in knowledge between the Appalachian counties (Counties 1 and 2) versus the non-Appalachian counties (Counties 3 and 4). Therefore, the data were combined and presented for the 4 counties (Table 3).

**Table 3.** Curriculum learning objectives and pre-/post-knowledge change.

Learning objectives	Assessment questions [*correct answer*]^a^	Percentage change in participant knowledge	*P* value
*Lesson: Fun with phytonutrients (n = 18)*			
At the conclusion of this lesson, participants will be able to: • Identify what phytonutrients are. • Identify where phytonutrients are found and the corresponding colors of fruits and vegetables. • Describe how phytonutrients protect the body against harmful effects of environmental pollution. • Understand the relationship between phytonutrients and pollution. • Learn the health benefits of consuming phytonutrients.	Phytonutrients (also known as phytochemicals, bioflavonoids, or polyphenols) are compounds found in [*plant-based foods*]^b^	+26.0	.01*
	Which of the following is a phytonutrient? [*anthocyanin*]^b^	+49.1	.001*
	Phytonutrients protect against the negative health effects of environmental pollution by doing which of the following? [*decreasing inflammation*]^c^	+20.7	.16
*Lesson: Healthy ways to flavor your food (n = 31)*			
At the conclusion of this lesson, participants will be able to: • Learn the difference between an herb and a spice. • Learn the health benefits of budget-friendly herbs and spices. • Shopping tips and meal ideas to use beneficial herbs and spices. • Understand how physical activity and certain spices can both help maintain glucose control.	Artificial flavorings are derived from [*chemicals*]^b^	+5.7	.31
	Physical activity in combination with the consumption of [*cinnamon*] may improve glucose control^b^	+20.2	.03*
	Herbs and spices protect against the negative health effects of environmental pollution by doing which of the following [*decreasing free radicals and oxidation*]^c^	+34.9	.01*
*Lesson: Fundamentals of fermented foods (n = 24)*			
At the conclusion of this lesson, participants will be able to: • Understand how fermented foods support the good bacteria in our bodies and how they support gut health. • Be familiar with common fermented foods. • Understand what probiotics and prebiotics are. • Define pollution and explain the link between environmental pollution and negative effects on health. • Understand the relationship between fermentation and pollution. • Learn how exercise supports gut health.	Probiotics are derived from [*live bacteria and yeasts within certain foods*]^b^	0.0	.71
	Which of the following is a fermented food? [*yogurt*]^b^	+44.2	.002*
	Fermented foods protect against the negative health effects of environmental pollution by doing which of the following? [*strengthening the immune system*]^c^	+20.7	.64
*Lesson: Getting to know GMOs (n = 27)*			
At the conclusion of this lesson, participants will be able to: • Define GMO and learn the history of GMOs. • Understand the relationship between GMOs, pollution, and pesticides. • Learn the pros and cons of GMOs. • Learn what foods are genetically modified.	GMOs must be approved for safety by the [*FDA*]^b^	+27.7	.003*
	In some cases, GMOs use [*less pesticides*]^b^	+38.6	.001*
	Genetic modification makes crops [*more useful*]^b^	+24.5	.02*
*Lesson: Picking out produce: all about organic and conventional foods (n = 24)*			
At the conclusion of this lesson, participants will be able to: • Differentiate conventional and organic labels. • Learn how to reduce exposure to pesticides. • Learn the pros and cons of organic and conventional foods. • Understand what genetically modified organisms are and what “all natural” can mean.	Pheromones or microbes that are sprayed on plants are considered [*biological-based*] pesticides^c^	+64.8	0.001*
	USDA Organic-certified foods may have been purposefully exposed to pesticides in the process of growing or raising a particular food [*true*]^c^	+12.0	.18
	Which of the following typically contains fewer pesticides? [*oranges*]^c^	−1.7	1.0
*Lesson: Cut down on environmental pollutants in your food (n = 29)*			
At the conclusion of this lesson, participants will be able to: • Learn why certain foods contain pollutants. • Learn ways to consume healthy fish. • Learn what arsenic is and where it is found. • Choose and cook healthy meat and dairy products.	A way we are exposed to environmental pollutants, such as polychlorinated biphenyls (PCBs) and mercury, is from [*eating contaminated foods*]^c^	+20.7	.03*
	Which of the following foods typically contains the highest concentration of arsenic? [*brown rice*]^c^	+32.8	.01*
	Which of the following foods is most likely to have the highest concentrations of PCBs? [*whole milk*]^c^	−1.0	1.0
*Lesson: Prevalent preservatives and safe storage of food (n = 25)*			
At the conclusion of the is lesson, participants will be able to: • Define food preservative. • Learn about different preservatives and which ones are safe. • Learn about the pros and cons of different food storage containers. • Determine whether certain drink bottles are considered safe.	All preservatives used in our food are currently approved by the FDA and are considered safe. [*true*]^c^	+30.0	.02*
	Which of the following storage methods is considered the safest? [*aseptic packaging*]^c^	+28.8	.01*
	BPA (bisphenol A) is a type of ______ associated with disrupting normal hormone function. [*plastic*]^c^	−5.6	.99
*Lesson: Deciding on a healthy drink (n = 28)*			
At the conclusion of this lesson, participants will be able to: • Learn the health effects of coffee. • Learn about different types of tea and their health benefits. • Learn all about water. • Differentiate sugary drinks. • Learn why milk is a good drink choice.	The American Heart Association recommends limiting sugar consumption to less than [*6*] teaspoons for women and [*9*] teaspoons for men^b^	+56.8	.0001*
	Drinking low-fat chocolate milk after moderate exercise has been associated with [*building muscle*]^b^	+47.1	.001*
	Coffee contains the phytonutrients, polyphenols, that act as [*antioxidants*]^b^	+52.1	.0003*
*Lesson: Nutritious nuts and seeds (n = 25)*			
At the conclusion of this lesson, participants will be able to: • Prioritize characteristics of healthy nuts and seeds when making a selection. • Recognize the relationship between the health effects of environmental pollutants and the protective properties of a nutritious diet. • Indicate proper storage techniques for nuts and seeds.	Nuts are from [*a hard-shelled dry fruit*]^b^	−24.1	.06
	A serving or recommended amount of nuts and seeds is [*1/3 cup*]^b^	+46.7	.001*
	Which nut or seed contains the highest level of omega-3 fatty acids?[*walnuts*]^b^	−11.9	.32

Abbreviation: GMOs, genetically modified organisms.

a All questions were presented using the multiple choice format with 4 choices per question unless otherwise noted in the table as a true/false.

b Pre- and post-lesson questions focused on food knowledge and healthy lifestyle knowledge-based information.

c Pre- and post-lesson questions focused on environmental pollutant knowledge.

* *P* ⩽ 0.05.

### Questionnaires

Of the 27 knowledge/awareness-based questions administered pre- and post-lesson, knowledge/awareness significantly improved for 17 questions. There were significant improvements in 50% of the 12 environmental pollution knowledge-based questions and 73% of the 15 food knowledge and healthy lifestyle questions (Table 3).

### Focus groups

Following their participation in the *Body Balance* series, respondents discussed their perception of environmental pollution and health while sharing examples of how their awareness, knowledge or behavior changed. All focus group participants were white women over the age of 55 years. Themes and representative quotes are listed below.

#### Environmental pollutants and environmental media of concern following Body Balance curriculum

When asked what comes to mind when they hear environmental pollution, participants named various environmental pollutants:

Well, you have to watch what you eat, what people put on food that you buy, the chemicals that are sprayed or whatever people call that.

and environmental media:

Garbage. You know it gets in the waters. Especially our creeks.

#### Participants’ level of concern about pollution increased following the lesson series

Environmental pollution concern increased significantly, 2.8 ± 1.2 to 4.7 ± 0.6 (5-point scale, *P* ⩽ 0.001). Participants shared the change was due to increased knowledge and awareness:

We became more knowledgeable of what the stuff was. We may have learned too much.

#### Implemented environmentally friendly practices and lifestyle choices to reduce exposure to pollution

When asked whether it ever crossed their minds that lifestyle choices can change how environmental pollution affects the body, most participants offered their environmental practices to protect the environment:

I try to recycle what I can recycle . . . .

When I go for a walk I take a trash bag sometimes so I can pick up other people’s trash . . .

Some participants shared food choices they made or recognized could be made to reduce exposure to environmental pollutants and preservatives/food additives in foods:

I make an effort to can my own foods and reuse the glass so I don’t have to keep buying more. And you control your nutrition, the ingredients.

As well, they mentioned behaviors they practiced before the lessons series to reduce their exposure to environmental pollution:

We just choose not to smoke.

. . . when the tobacco barns are up you try not to get outside as much.

What you put on, what you spray around the soil, (inaudible), we grew up with a well and we were always protective that nothing would be near the well because that was our water supply.

#### Implemented healthy lifestyle choices

Participants named specific examples of behavior changes they made as a result of participating in the *Body Balance* curriculum. Lifestyle changes included trying to eat more fruits and vegetables (22%), reading food labels (44%) to garner information pertaining to sugar and sodium content, to determine where food was packaged or distributed, and ingredients. Participants reported consuming fewer sugar-sweetened beverages (11%) and less fast food and processed foods, more blueberries, a greater quantity and variety of nuts to salads and a greater variety of vegetables; avoided purchasing deli meats because of high sodium content; incorporated more fresh produce into diet because of too much sodium in canned vegetables; incorporated more fermented foods into diet; stopped re-heating foods in plastic containers and started checking numbers on their storage containers; and began cooking more meals (1%):

Reading the labels. Yes, that was a big one.

. . . I don’t buy a lot of cans, most of what I buy now is frozen or fresh. I have changed that. Cause the frozen, they don’t put hardly anything in it.

I changed to a glass bowl when I heat something up.

#### Increased nutrition knowledge and knowledge of pollution sources

The nutrition knowledge gained was primarily in relation to information garnered from the food label particularly the ingredient list, which helped discern which foods contained artificial flavors and colorings as well as sugar and sodium content:

I had never read a label, but I was so surprised. Drinks especially, are so bad for you. They just had so much sugar.

Knowledge pertaining to environmental pollution centered on foods or packaging being sources of pollutants or contaminants that negatively affect health as well as foods that contained components beneficial to health:

I changed to a glass bowl when I heat something up.

Learning about that phytonutrients. That was a new word and now I’ve seen it on foods, cans and things.

I didn’t realize fermented foods could help you get rid of pollutants.

Participants learned that organic produce options may not be the best choice for them because they did not stay preserved as long as conventional foods. They learned that organic foods may also contain pesticides and the level of pesticides sprayed on conventional or organic foods is regulated and reasoned that it is likely safe:

Even if it’s not organic they do still have limits to the pesticides that are going on anyway.

#### Barriers to implementing lifestyle changes

Participants recognized a number of barriers related to implementing changes, but did not specifically mention barriers in relation to decreasing exposure to environmental pollution:

. . . based on your resources and your availability for transportation.

“I think there’s a way to do healthy lifestyle if you choose.” “Yes.” “It’s the choosing that matters.”

yea, it’s money. Well, accessibility but also money.

In our area it’s hard to find a place to exercise unless you exercise at home or walk on the road where we live at . . .

However, as one participant expressing efficacy-related frustration put it, others need to “cut down on environmental pollutants. Other than washing our food, it is what it is.”

## Discussion

The purpose of this study was to determine whether EHL levels and self-reported protective food-related behaviors improved among community members following participation in the FCS Extension agent–led *Body Balance: Protect Your Body from Pollution with a Healthy Lifestyle* curriculum. The *Body Balance* curriculum is distinctive because it bridges the concepts of health promotion and environmental health and was delivered by a trusted community member. Pilot data demonstrated an increase in both nutrition and pollution knowledge among study participants. Moreover, focus group participants highlighted several specific behavior changes they made as a result of their participation in the *Body Balance* curriculum. The FCS agent was reported to be an important component in participants partaking of healthier lifestyle activities.

The *Body Balance* curriculum is an environmental health education curriculum. As expected from such a curriculum, we saw increased EHL levels, specifically awareness and knowledge regarding environmental pollution, food, and dietary-related strategies.^51^ The significant increase in knowledge for 63% of questions asked from pre- to post-lesson indicates poor baseline knowledge pertaining to healthy behaviors, pollution, and exposure to pollution. The focus group data were supportive of the quantitative data as participants specifically stated examples of what they learned from the *Body Balance* curriculum. *Body Balance* also increased participant awareness of dietary sources of environmental pollution, the negative health effects of pollution, and various food strategies that influence exposure to pollution and enhance poor nutrition. The low levels of baseline knowledge align with expectations based on the literatures of both health literacy^52^ and EHL.^53^ Furthermore, previous research has shown rural Appalachians to have poor nutrition knowledge.^54^ The adapted Bloom’s taxonomy conceptual model of EHL is useful to explain the degree of learning by *Body Balance* participants.^53^ The *Body Balance* curriculum helped participants reach the “recognition” stage of learning and understanding that lifestyle activities, food, and food preparation strategies can influence their exposure to pollution as well as the effect of pollution on their health. The fact that focus group participants did not voice dietary strategies as a method of protecting their health from environmental pollution, but self-reported making behavior changes because of an increase in knowledge and their FCS agent emphasizing the concepts, demonstrates that participants did not reach the EHL stage of “understanding” to internalize how protection occurs with lifestyle strategies.

The self-reported behavior changes of 44% of focus group participants was a notable finding. During the focus groups, participants cited 2 factors that contributed to their self-reported behavior change; knowledge gain and the FCS agent as reasons why they made changes. Having the FCS agent deliver the curriculum was critical in our study as they likely served as “agents of change.” Because FCS agents live in the communities they serve, they establish trusting, long-term relationships which are all key characteristics of “agents of change.”^55^ The success of “agents of change” is directly related to their effort in connecting with target audiences, which FCS agents in our study clearly did. Furthermore, the self-reported positive behavior changes may have occurred because of the curriculum providing nutrition education, healthy recipes, food samples, and hands-on activities to reinforce the messages. Previous research has shown an association between increased knowledge and improved dietary intake, specifically fruit and vegetable consumption, following a nutrition education series.^54^ Furthermore, shifting the health message away from shaming and personal responsibility can also influence behavior.^56,57^ Researchers developed the *Body Balance* curriculum to focus on exposure-centric rather than disease-centric behaviors. The key message delivered by agents throughout the lesson series encouraged consumption of a nutritious diet to protect health from environmental pollution rather than focusing on a particular condition. By not focusing on stigmatized chronic diseases, a health program focused on mitigating the effects of pollution avoids the shame and perceived personal responsibility of those diseases. This enhances the efficacy of a health program.^56,57^

In general, the FCS Extension agent–delivered *Body Balance* curriculum was an effective mechanism to raise baseline EHL among participants when agents shared information related to nutrition’s potential to modulate the toxicity of environmental pollution as an Extension curriculum. The middle- to older-aged audience of this pilot study was an appropriate one to engage not only because of where they lived but because living longer has exposed them to more environmental contaminants, and with age the detoxification capacity of the liver and kidney have declined to put older adults at greater risk of experiencing pronounced negative health effects of environmental contaminants.^58^ With Kentuckians potentially being exposed to a variety of environmental pollutants across the Commonwealth, the *Body Balance* curriculum is an appropriate curriculum for any of the FCS Extension agents across Kentucky to implement in their county. As with any extension curriculum, the Extension agent can choose which lessons are most appropriate for their community members and present them in a manner that resounds with their audience. Therefore, *Body Balance* is transferable to other settings and demographics other than those included in the pilot study. There were several advantages of having an FCS agent deliver the *Body Balance* curriculum that fostered the increase in EHL. First, the curriculum did not have to be delivered by CEC personnel thus preserving CEC resources and allowing flexibility in curriculum deliver, and second, the *Body Balance* curriculum was developed specifically for use by FCS agents, making it easily implementable within the train-the-trainer system of FCS Cooperative Extension.

The study had limitations. Researchers conducted the focus groups in the aftermath of forest fires throughout Kentucky and Tennessee. With smoke visible from many of the participating counties, the fires potentially raised the immediate salience of air quality concerns.^59^ Implementation of the *Body Balance* curriculum varied by county as each agent exercised discretion regarding the frequency of and logistical arrangements for lessons. Not all participants were able to attend all 9 lessons. Agents may have emphasized the material with which they were more familiar and comfortable. Deployment of the train-the-trainer model, however, brought stability in content delivery. The pilot study included a small number of participants and used a convenience sample, but the recruitment and delivery of *Body Balance* mimicked the typical process agents follow when offering an extension curriculum. We did not conduct post-evaluation assessment of knowledge retention and maintenance of behavior change, but the focus groups were conducted 3 months following the conclusion of the curriculum and participants were reporting at that time they were still implementing certain changes. Also noted is that focus group data reflect self-reported behavior change, in a room of peers, to an unfamiliar researcher. As such, results should be interpreted with care. This study occurred in rural Kentucky and results might not be transferable to other Kentucky counties.

## Conclusions

Pilot study results indicate that white, female, middle/older-aged Kentuckians who participated in the *Body Balance* FCS Extension curriculum significantly increased their knowledge and awareness of healthy behaviors, pollution, and exposure to pollution. Following the lesson series, participants understood diet and exercise to be the cornerstone of a healthy lifestyle, but they did not vocalize that healthy lifestyles may mitigate the negative health effects related to environmental pollution. However, participants did self-report behavior changes arising from increased knowledge and support by their FCS agent. Findings indicate participants achieved the “recognition” stage of EHL,^53^ learning and understanding that physical activities, food, and food preparation strategies can affect both their risks of exposure to pollution and the effects of such pollution on human health. The FCS agents themselves were key to the success of the pilot program, serving as both knowledge brokers and “agents of change.” There is a need for a frequent, consistent, and widespread delivery and testing of messages about nutrition and environmental pollution to continue assessing how best to propel people from EHL recognition stage to the action stage.

## References

[1] Khansari N Shakiba Y Mahmoudi M. Chronic inflammation and oxidative stress as a major cause of age-related diseases and cancer. Recent Pat Inflamm Allergy Drug Discov. 2009;3:73–80. 19149749 10.2174/187221309787158371

[2] Roberts C Barnard R. Effects of exercise and diet on chronic disease. J Appl Physiol. 2005;98:3–30. 15591300 10.1152/japplphysiol.00852.2004

[3] Rappaport SM Smith MT. Environment and disease risks. Science. 2010;330:460–461. 20966241 10.1126/science.1192603 PMC4841276

[4] Hennig B Ettinger AS Jandacek RJ et al Using nutrition for intervention and prevention against environmental chemical toxicity and associated diseases. Environ Health Perspect. 2007;115:493–495. 17450213 10.1289/ehp.9549 PMC1852675

[5] Petriello MC Newsome BJ Dziubla TD Hilt JZ Bhattacharyya D Hennig B. Modulation of persistent organic pollutant toxicity through nutritional intervention: emerging opportunities in biomedicine and environmental remediation. Sci Total Environ. 2014;491–492:11–16. 10.1016/j.scitotenv.2014.01.109 PMC4077968 24530186

[6] Bernard A Hermans C Broeckaert F De Poorter G De Cock A Houins G. Food contamination by PCBs and dioxins. Nature. 1999;401:231–232. 10499576 10.1038/45717

[7] Guallar E Sanz-Gallardo MI van’t Veer P et al Mercury, fish oils, and the risk of myocardial infarction. N Engl J Med. 2002;347:1747–1754. 12456850 10.1056/NEJMoa020157

[8] Huq SI Joardar JC Parvin S Correll R Naidu R. Arsenic contamination in food-chain: transfer of arsenic into food materials through groundwater irrigation. J Health Popul Nutr. 2006;24:305–316. 17366772 PMC3013251

[9] Hennig B Ormsbee L McClain CJ et al Nutrition can modulate the toxicity of environmental pollutants: implications in risk assessment and human health. Environ Health Perspect. 2012;120:771–774. 22357258 10.1289/ehp.1104712 PMC3385446

[10] Liu RH. Health-promoting components of fruits and vegetables in the diet. Adv Nutr. 2013;4:384S-392S. 23674808 10.3945/an.112.003517 PMC3650511

[11] Aragno M Mastrocola R. Dietary sugars and endogenous formation of advanced glycation endproducts: emerging mechanisms of disease. Nutrients. 2017;9:E385. 28420091 10.3390/nu9040385 PMC5409724

[12] Bengmark S. Advanced glycation and lipoxidation end products—amplifiers of inflammation: the role of food. J Parenter Enteral Nutr. 2007;31:430–440. 10.1177/0148607107031005430 17712153

[13] Freitas-Simoes TM Ros E Sala-Vila A. Nutrients, foods, dietary patterns and telomere length: update of epidemiological studies and randomized trials. Metabolism. 2016;65:406–415. 26975532 10.1016/j.metabol.2015.11.004

[14] Nerín C Aznar M Carrizo D. Food contamination during food process. Trends Food Sci Technol. 2016;48:63–68.

[15] Department for Environmental Protection. Fiscal Year 2018 Annual Report. Frankfort, KY: Commonwealth of Kentucky; 2018.

[16] United States Environmental Protection Agency. Superfund National Priorities List (NPL) Sites—by State. https://www.epa.gov/superfund/national-priorities-list-npl-sites-state.Up-dated2018.

[17] Commonwealth of Kentucky Energy and Environment Cabinet. EPA Toxic Release Inventory (TRI): Kentucky At-A-Glance—2016 Reporting Year. Frankfort, KY: Department for Environmental Protection; 2016.

[18] United States Environmental Protection Agency. 2016 TRI factsheet: state—Kentucky. https://iaspub.epa.gov/triexplorer/tri_factsheet.factsheet_forstate?&pstate=KY&pyear=2016&pParent=NAT.Up-dated2018. Accessed January 30, 2019.

[19] American Lung Association. Toxic air pollutants. https://www.lung.org/our-initiatives/healthy-air/outdoor/air-pollution/toxic-air-pollutants.html.Up-dated2019. Accessed January 30, 2019.

[20] United States Environmental Protection Agency. Ground water nad drinking water: national primary drinking water regulations. https://www.epa.gov/ground-water-and-drinking-water/national-primary-drinking-water-regulations.Up-dated2018. Accessed January 30, 2019.

[21] Kentucky Geological Survey. Water Fact Sheet. Lexington, KY: University of Kentucky; 2014.

[22] United States Environmental Protection Agency. Private drinking water wells. https://www.epa.gov/privatewells.Up-dated2018. Accessed January 30, 2019.

[23] Agency for Toxic Substances & Disease Registry. Toxic substances portal: trichloroethylene (TCE). https://www.atsdr.cdc.gov/substances/toxsubstance.asp?toxid=30.Up-dated2011. Accessed January 30, 2019. 38011294

[24] Hoover AG Defining environmental health literacy. In: Finn S O’Fallon LR, eds. Environmental Health Literacy. New York, NY: Springer; 2019:3–18.

[25] Ramirez-Andreotta MD Brody JG Lothrop N Loh M Beamer PI Brown P. Improving environmental health literacy and justice through environmental exposure results communication. Int J Environ Res Public Health. 2016;13:E690. 27399755 10.3390/ijerph13070690 PMC4962231

[26] Derrick CG Miller JSA Andrews JM. A fish consumption study of anglers in an at-risk community: a community-based participatory approach to risk reduction. Public Health Nurs. 2008;25:312–318. 18666936 10.1111/j.1525-1446.2008.00711.x

[27] Sullivan J Using Augusto Boal’s theatre of the oppressed in a community-based participatory research approach to environmental health literacy. In: Finn S O’Fallon LR, eds. Environmental Health Literacy. New York, NY: Springer; 2019:285–314.

[28] Finn S O’Fallon LR. The emergence of environmental health literacy—from its roots to its future potential. Environ Health Perspect. 2015;125:495–501. 26126293 10.1289/ehp.1409337 PMC5382009

[29] Foundation UH. America’s health rankings 2018 annual report. https://www.americashealthrankings.org/learn/reports/2018-annual-report.Up-dated2018. Accessed January 30, 2019.

[30] Landrigan PJ Fuller R Acosta NJR Edeyi O Arnold R Basu N. The Lancet commission on pollution and health. Lancet. 2018;391:462–512. 29056410 10.1016/S0140-6736(17)32345-0

[31] United States Census Bureau. American fact finder. https://factfinder.census.gov/faces/nav/jsf/pages/index.xhtml. Accessed January 30, 2019.

[32] Foundation for a Healthy Kentucky. Tracking health values. www.kentuckyhealthfacts.org. Up-dated 2008. Accessed December 10, 2017.

[33] Henry J Kaiser Family Foundation. State health facts: number of cancer deaths per 100,000 population. https://www.kff.org/other/state-indicator/cancer-death-rate-per-100000/?currentTimeframe=0&sortModel=%7B%22colId%22:%22Location%22,%22sort%22:%22asc%22%7D. Up-dated 2016. Accessed January 30, 2019.

[34] Henry J Kaiser Family Foundation. State health facts: diabetes. https://www.kff.org/other/state-indicator/adults-with-diabetes/?currentTimeframe=0sortModel=%7B%22colId%22:%22Location%22,%22sort%22:%22asc%22%7D. Up-dated 2017. Accessed January 30, 2019.

[35] Henry J Kaiser Family Foundation. State health facts: number of deaths due to diseases of the heart per 100,000 population. https://www.kff.org/other/state-indicator/number-of-deaths-due-to-diseases-of-the-heart-per-100000-population/?currentTimeframe=0&sortModel=%7B%22colId%22:%22Location%22,%22sort%22:%22asc%22%7D. Up-dated 2016. Accessed January 30, 2019.

[36] USDA. Rural-urban continuum codes: documentation. https://www.ers.usda.gov/data-products/rural-urban-continuum-codes/documentation/. Up-dated 2018. Accessed January 30, 2019.

[37] University of Wisconsin Population Health Institute. County health rankings https://uwphi.wiscweb.wisc.edu/wp-content/uploads/sites/316/2017/11/WCHR_2017_Rankings.pdf. Up-dated 2017.

[38] Dunn K Gaetke L Stephenson T Brewer D. Older adults’ perception of nutrition being protective against the detrimental health effects of environmental pollution. J Ext. 2017;55:4RIB7. PMC5697776 29176912

[39] American Association of Family & Consumer Sciences. What is FCS? https://www.aafcs.org/about/about-us/what-is-fcs. Accessed December 15, 2017.

[40] Haughton B. Applying the socio-ecological model to nutrition issues that promote health and prevent disease. Fam Community Health. 2006;29:3–4.

[41] Centers for Disease Control and Prevention. Social ecological model. https://www.cdc.gov/cancer/nbccedp/sem.htm Up-dated 2013. Accessed January 20, 2019.

[42] U.S. Department of Health and Human Services. 2015–2020 Dietary Guidelines for Americans. https://health.gov/dietaryguidelines/2015/resources/2015-2020_dietary_guidelines.pdf. Up-dated December, 2015.

[43] Programme UND. World Energy Assessment: Energy and the Challenge of Sustainability. London, England: World Energy Council; 2000.

[44] Council SWKED. Agribusiness. http://southwesternky.com/local-business/agribusiness/. Up-dated 2019. Accessed January 30, 2019.

[45] Atlas S Overview of the United States. https://statisticalatlas.com/United-States/Overview. Up-dated 2018. Accessed January 30, 2019.

[46] Spengler JD Koutrakis P Dockery DW Raizenne M Speizer FE. Health effects of acid aerosols on North American children: air pollution exposures. Environ Health Perspect. 1996;104:492–499. 8743436 10.1289/ehp.96104492 PMC1469351

[47] Aneja VP Isherwood A Morgan P. Characterization of particulate matter (PM10) related to surface coal mining operations in Appalachia. Atmos Environ. 2012;54:496–501.

[48] Ray JA O’Dell PW Moody JR Blanset JM Blair RJ. Identification and Prioritization of Karst Groundwater Basins in Kentucky for Targeting Resources for Nonpoint Source Pollution Prevention and Abatement. Frankfort, KY: Kentucky Division of Water; 2005.

[49] Kentucky Geological Survey. Water quality.http://www.uky.edu/KGS/water/library/gwatlas/Washington/Waterquality.htm. Up-dated 2018. Accessed January 30, 2019.

[50] Butler MO. Evaluation: A Cultural Systems Approach. Walnut Creek, CA: Left Coast Press; 2015.

[51] Guidotti TL. Communication models in environmental health. J Health Commun. 2013;18:1166–1179. 23898914 10.1080/10810730.2013.768725

[52] Nutbeam D. The evolving concept of health literacy. Soc Sci Med. 2008;67:2072–2078. 18952344 10.1016/j.socscimed.2008.09.050

[53] Finn S O’Fallon L. The emergence of environmental health literacy-from its roots to its future potential. Environ Health Perspect. 2017;125:495–501. 26126293 10.1289/ehp.1409337 PMC5382009

[54] Tessaro I Rye S Parker L Mangone C McCrone S. Effectiveness of a nutrition intervention with rural low-income women. Am J Health Behav. 2007;31:35–43. 17181460 10.5555/ajhb.2007.31.1.35

[55] Center for Community Health Development. Identifying targets and agents of change: who can benefit and who can help. http://ctb.ku.edu/en/table-of-contents/analyze/where-to-start/identify-targets-and-agents-of-change/main. Up-dated 2017. Accessed December 15, 2017.

[56] Earnshaw VA Quinn DM Park CL. Anticipated stigma and quality of life among people living with chronic illnesses. Chronic Illn. 2012;8:79–88. 22080524 10.1177/1742395311429393 PMC3644808

[57] Robinson B Coveleski S. Don’t say that to ME: opposition to targeting in weight-centric intervention messages. Health Commun. 2018;33:139–147. 27911089 10.1080/10410236.2016.1250189

[58] Geller AM Zenick H. Aging and the environment: a research framework. Environ Health Perspect. 2005;113:1257–1262. 16140638 10.1289/ehp.7569 PMC1280412

[59] Brannock L Searcy C. Fighting Kentucky’s Wildfires: A Great Coordinated Effort. Frankfort, KY: Kentucky Energy and Environment Cabinet: Kentucky Energy and Environment; 2016.

